# Association between socioeconomic status and severity of oral epithelial dysplasia using a Taiwanese Nationwide Oral Mucosal Screening Program: a retrospective analysis

**DOI:** 10.1186/s12903-022-02084-7

**Published:** 2022-03-04

**Authors:** Tien-En Chiang, Yu-Chun Lin, Chi-Tsung Wu, Sheng-Tang Wu, Yuan-Wu Chen

**Affiliations:** 1 Division of Oral and Maxillofacial Surgery, Tri-Service General Hospital, No. 325, Cheng-Kung Rd., Sec. 2, Neihu District, Taipei City, 11490 Taiwan, ROC; 2 School of Dentistry, National Defense Medical Center, No. 161, Sec. 6, Minquan E. Rd., Neihu Dist., Taipei City, 11490 Taiwan, ROC; 3 Department of Pathology, Tri-Service General Hospital, No. 325, Sec. 2, Chenggong Rd., Neihu District, Taipei City, 11490 Taiwan, ROC; 4 Division of Urology, Department of Surgery, Tri-Service General Hospital, National Defense Medical Center, No. 161, Sec. 6, Minquan E. Rd., Neihu Dist., Taipei City, 11490 Taiwan, ROC

**Keywords:** Oral Epithelial Dysplasia, Oral Potentially Malignant Disorders, Taiwanese Nationwide Oral Mucosal Screening Program, Socioeconomic Status

## Abstract

**Background:**

The study aimed to investigate the association between socioeconomic status and severity of oral epithelial dysplasia (OED) using current data from the Taiwanese Nationwide Oral Mucosal Screening Program (TNOMSP).

**Methods:**

This retrospective analysis was conducted in the Department of Oral and Maxillofacial Surgery at a general hospital in Taipei, Taiwan. A total of 134 participants were analysed from a previous study database of 150 patients. The inclusion criteria included age > 20 years and a history of either tobacco or betel nut use. Background information, including para-habits such as betel and tobacco use, was analysed using the Pearson chi-square (χ^2^) test; furthermore, the correlation of background information with OED severity was investigated using logistic regression (mild or moderate/severe).

**Results:**

High school education level (*P* < 0.001), poor self-awareness (*P* = 0.002), current betel use (*P* < 0.001), and tobacco use (*P* = 0.003) were highly correlated with moderate- and severe OED (*P* < 0.05). The odds ratio (OR) of education status above senior high school was 0.03 (95% confidence interval [CI] 0.01–0.15, *P* < 0.001*), while that o*f junior high school was 1. Current betel chewing (OR 6.57 [95% CI 1.17–37.0], *P* = 0.033) was significantly associated with OED severity compared with never or ex-use of betel.

**Conclusions:**

We found a strong correlation between the severity of OED and current betel use and low education status. The current study revealed that the socioeconomic status, poor self-awareness, and para-habit history of the patients with OED should be evaluated to identify high-risk individuals using TNOMSP.

## Background

Histopathological grading of oral epithelial dysplasia (OED) is predictive for oral potentially malignant disorders (OPMDs) [1]. The proportion of OED associated with malignant transformation is approximately 2.2–38.1% [2, 3]. The histopathological manifestation of oral potentially malignant disorders may be revealed as hyperkeratosis or hyperplasia, to various degrees of dysplasia, categorised as mild, moderate, and high grade according to the severity of cell atypia and epithelial involvement [4]. Therefore, the precision of conventional oral examination (COE) and early diagnosis of OED is crucial for preventing malignant transformation [5].

COE for oral mucosa screening aims to reduce mortality in malignancy and guide preventive interventions for high-risk groups [6]; thus, the definitive diagnosis is crucial. Warnakulasuriya et al. had proposed the term OPMDs and recently updated it as “any oral mucosal abnormality that is associated with a statistically increased risk of developing oral cancer.” This classification of disorders previously included leukoplakia, proliferative verrucous leukoplakia, erythroplakia, submucosal fibrosis, palatal lesions in reverse smokers, oral lichen planus, acinic keratosis, oral lupus erythematosus, and dyskeratosis congenita. Recently, oral lichenoid lesion and oral graft vs. host disease were added, and oral epidermolysis bullosa was removed from the classification due to limited evidence [7, 8].

The major risk factors for OED in patients with OPMDs vary across geographical regions. In Southeast Asia, two well-established risk factors are recognised: betel quid chewing and tobacco smoking [9, 10]. In Taiwan, tobacco smoking and betel quid chewing have been shown to increase the risk of leukoplakia and malignant transformation of OED [11, 12]. Nevertheless, the association between socioeconomic status and the development of malignancy involves poor health education, unfavourable working environments, and many others, which all contribute to malignancy development by complex interactions in society; most importantly, risk behaviours are often seen in the low socioeconomic status groups [13]. In the Asia region, these risk behaviours are notoriously known as betel chewing and smoking; therefore, these serious issues have been designed into effective health policy programmes for the high-risk group, implemented in several countries [14].

COE is a relatively easy procedure used to identify oral lesions [15]. The Taiwanese government has supported oral screening programs for many years [16], and the Taiwanese Nationwide Oral Mucosal Screening Program (TNOMSP) is principally developed and documented in a specific survey [17]. This survey gathers specific background information and para-habits of high-risk individuals. However, a more conclusive diagnosis can be obtained from a specialist after referral from primary health providers such as general dentists or family medicine physicians. By definition, in the referral programme, a specialist such as an oral and maxillofacial surgeon or otorhinolaryngology surgeon, who can provide treatment, is required to perform the needed biopsy. Additionally, a pathologist is required to confirm and document the OED stage from the biopsy; and, according to the current recommendation, a follow-up system for recall and monitoring in high-risk individuals with OPMD and OED should be arranged for effective management [1]. In this retrospective study, we analysed a group of participants documented in the TNOMSP and investigated the association between the socioeconomic status and severity of OED.

## Methods

A retrospective analysis was conducted in the Department of Oral and Maxillofacial Surgery at a general hospital in Taipei, Taiwan, to investigate the association between the socioeconomic status and severity of OED using current data from the TNOMSP.

The data were collected within 12 months, from January 1st to December 31st, 2018. A total of 134 participants were analysed from a previous study database of 150 patients; 10 patients were excluded due to their refusal to participate, and 6 patients were excluded as the study design was limited to Han Chinese only. The study was conducted in the Department of Oral and Maxillofacial Surgery at the Tri-service General Hospital (TSGH) of National Defense Medical Center (NDMC), Taipei, Taiwan.

This was a pseudo-anonymised secondary data study; hence, there was no direct patient or public involvement. The study was approved by the institutional review board of TSGH (1-107-05-010). The current study was reported in conformance with the STROBE guidelines.

### Inclusion and exclusion criteria

Inclusion criteria:

1. Patient age > 20 years.

2. History of tobacco use, current or past.

3. History of betel nut use, current or past.

Exclusion criteria:

1. Lack of proper informed consent of participation.

2. Non-Han Chinese.

3. Incomplete background information record from the TNOMSP form.

4. Incomplete histopathological diagnosis.

### Clinical examination procedure and TNOMSP

**Fig. 1:**
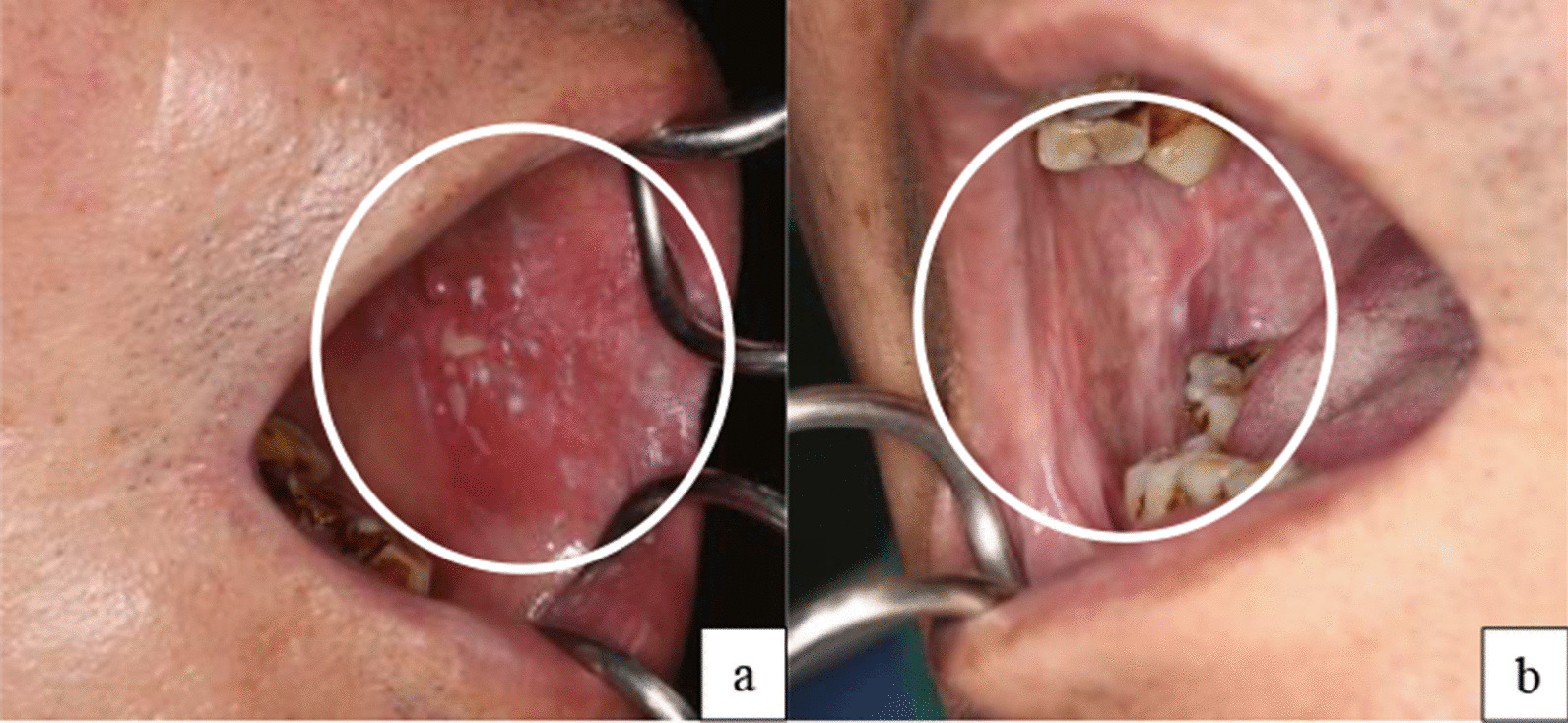
Study participants recruited from the TNOMSP study with Para-habits of Tobacco or Betel use. **a** OPMDs diagnosis: Left buccal erythroleukoplakia with current Betel chewing (Noted dental attrition and staining). **b** OPMDs diagnosis: Oral Submucous fibrosis with current Betel chewing (Noted dental attrition and staining). OPMD: Oral potentially malignant disorders, TNOMSP: Taiwanese Nationwide Oral Mucosal Screening Program

**Fig. 2:**
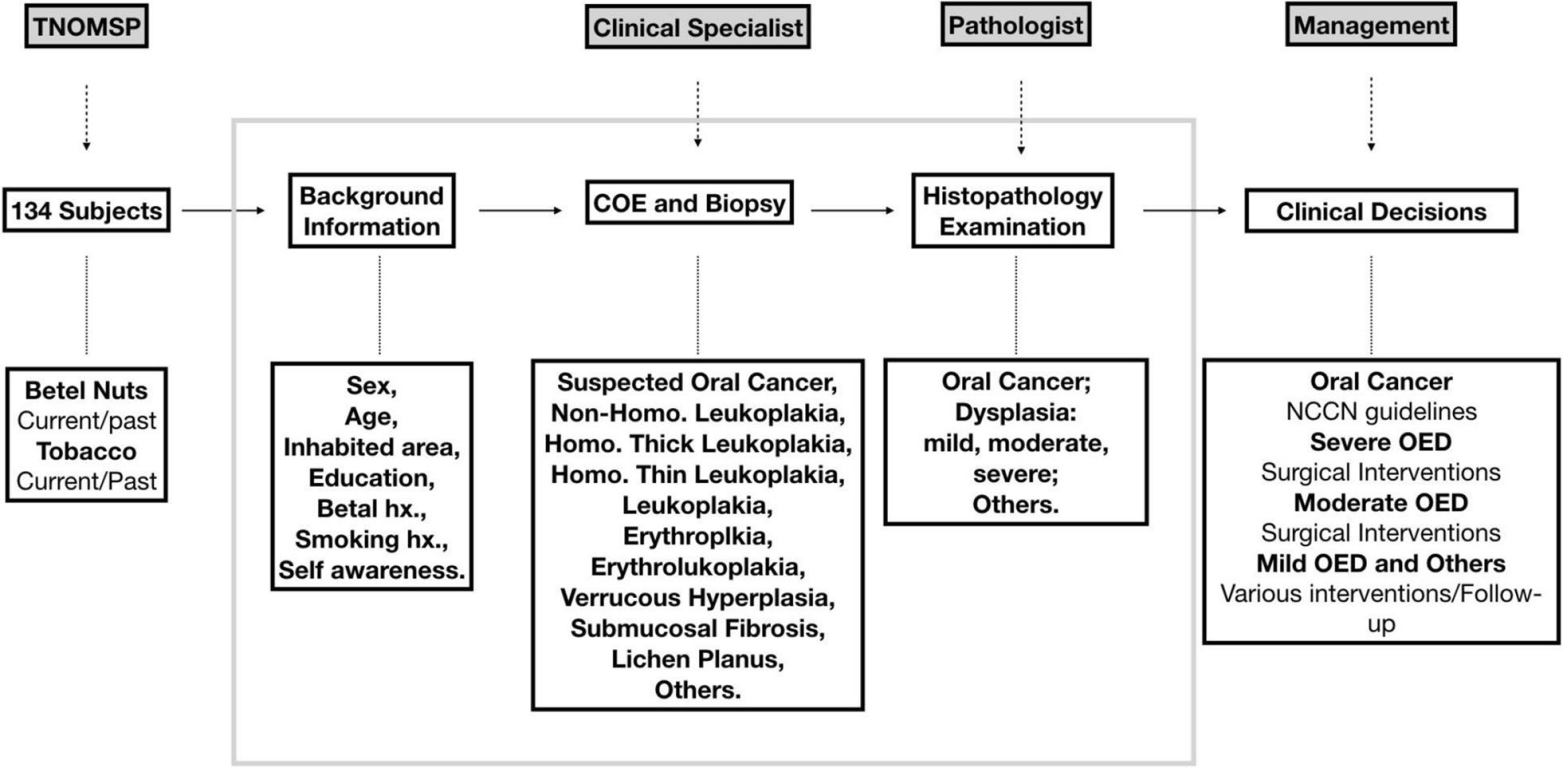
Subjects selected during the clinical examination procedure of TNOMSP

**Fig. 3:**
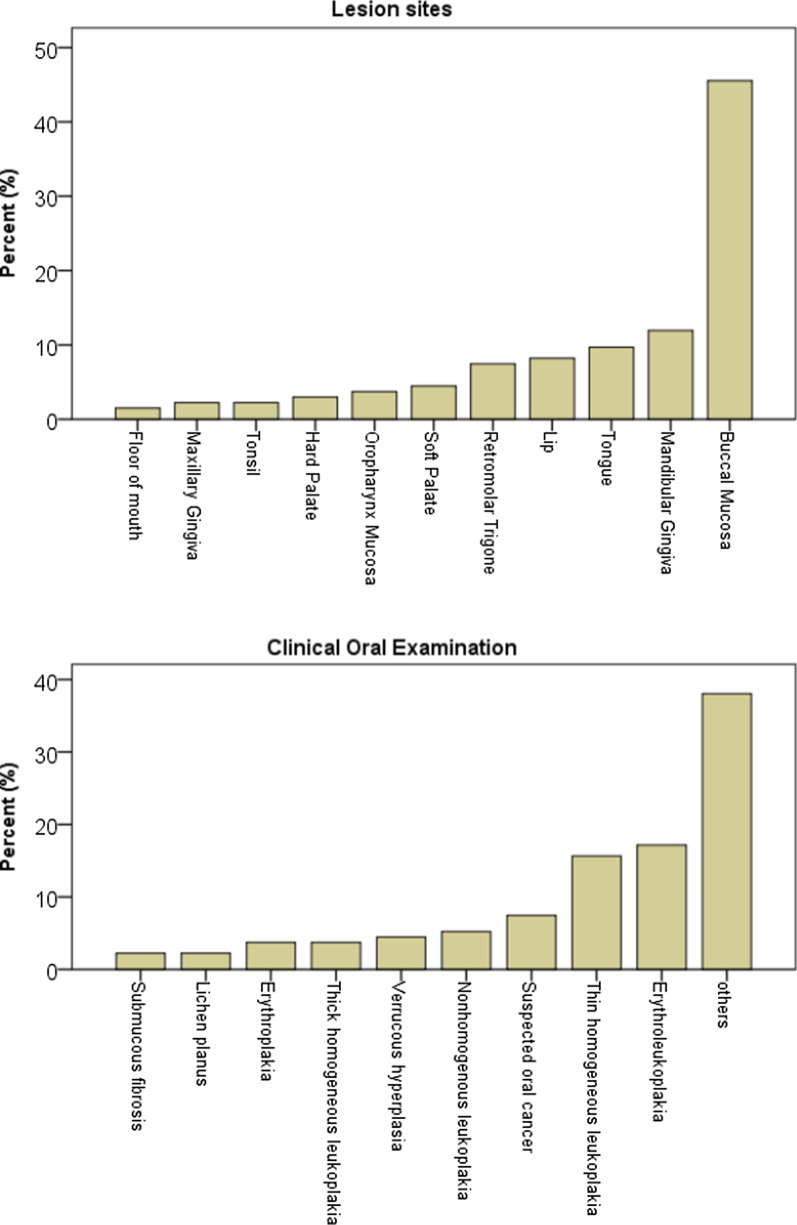
Distribution of clinical characteristics of OPMDs, oral cancer, others, and lesion sites. OPMD: Oral potentially malignant disorder

The participants were recruited from a previous study involving TNOMSP, developed by the Health Promotion Administration of the Ministry of Health and Welfare and commonly used for oral cancer and OPMD screening in Taiwan. The TNOMSP collects the following background information: name, sex, contact information, age, race, inhabited area, education, history of betel nut and tobacco use, and self-awareness. Personally identifiable information, such as name and contact information, was substituted with numbers. A clinical diagnosis of either OPMDs, suspected oral cancer, or others was obtained from a certified clinical specialist (oral and maxillofacial surgeon) (Fig. 1), who also recorded the lesion sites (Fig. 3). The OPMDs evaluated in the TNOMSP included non-homogenous leukoplakia, homogenous thick leukoplakia, leukoplakia, erythroplakia, erythroleukoplakia, verrucous hyperplasia, submucosal fibrosis, lichen planus, suspected oral cancer, and others. A clinical specialist performed oral lesion biopsy followed by a pathological diagnosis as mild-, moderate-, or severe dysplasia, oral cancer, or others. Finally, various interventions or follow-up visits were recommended for patients with mild dysplasia, and those with moderate and severe dysplasia were indicated for surgical interventions. Several other clinical decisions were made according to the provided guidelines in the institution conducting the study (Fig. 2).

### Histopathological data

The study participants agreed to participate and signed a standard informed consent form before biopsy was performed at TSGH. Each sample biopsy underwent histopathological examination by a pathologist. The presence of dysplasia was graded using the World Health Organization (WHO) three-tier system of mild, moderate, and severe dysplasia, oral cancer, or other diagnoses in the biopsy specimen. This was recorded in a report from the pathology department at TSGH, approved stepwise by two pathologists, if dispute the third pathologist for confirmation; the microscopic pictures were randomly selected and presented in our joint departmental meetings.

### Statistical analysis

All statistical analyses were performed using SPSS version 22.0.0. (IBM Corp., Armonk, NY, USA). The responses were coded as numeric to facilitate data entry. The results were analysed using a bar chart for clinical diagnosis of OPMDs, suspected oral cancer, other diseases, and lesion sites. The Pearson chi-square (χ^2^) test, and odds ratios (ORs) and 95% confidence intervals (95% CIs) were computed to determine any association between the participants’ characteristics and OED, which categorised the histopathologic reports into two groups mild or moderate and severe dysplasia after oral cancer and others diseases were excluded with the level of significance set as two-tailed *P* < 0.05.

## Results

### Participants’ demographics

A total of 134 participants out of 150 individuals in the original TNOMSP were collected in the present study (Table 1). The majority of our presented participants were male (n = 117, 87.3% vs. female: n = 17, 12.7%). Regarding education status, 103 participants (76.9%) reached either senior high school level or above. The number of participants residing in the capital (Taipei) and non-capital (Majority of New Taipei city surrounding Taipei city) areas were evenly distributed (n = 67, 50.0% vs. n = 67, 50.0%, respectively). Additionally, most of the participants (n = 114, 85.1%) were unaware of the lesions. Regarding para-habits, 36.6% of the participants were current betel users, while 53.8% were current smokers.

**Table 1:** Demographic variables and histopathology diagnosis using TNOMSP

	Histopathology			
	All	Dysplasia	Cancer	Others
	n(%)	n(%)	n(%)	n(%)
	n = 134	n = 70	n = 6	n = 58
Age	56.55 ± 12.93	58.71 ± 11.77	62.45 ± 13.07	53.34 ± 13.7
Gender				
Female	17 (12.7%)	13 (18.6%)	1 (16.7%)	3 (5.2%)
Male	117 (87.3%)	57 (81.4%)	5 (83.3%)	55 (94.8%)
Education				
Junior high school	31 (23.1%)	20 (28.6%)	3 (50.0%)	8 (13.8%)
Senior high school	55 (41.1%)	25 (35.7%)	1 (16.7%)	29 (50.0%)
University	48 (35.8%)	25 (35.7%)	2 (33.3%)	21 (36.2%)
Residing				
Capital	67 (50.0%)	33 (47.1%)	1 (16.7%)	33 (56.9%)
Others	67 (50.0%)	37 (52.9%)	5 (83.3%)	25 (43.1%)
Self-awareness				
No	114 (85.1%)	63 (90.0%)	0 (0%)	51 (87.9%)
Yes	20 (14.9%)	7 (10.0%)	6 (100%)	7 (12.1%)
Betel history				
Never used	49 (36.6%)	27 (38.6%)	2 (33.3%)	20 (34.5%)
Ex-users	36 (26.8%)	12 (17.1%)	2 (33.3%)	22 (37.9%)
Current users	49 (36.6%)	31 (44.3%)	2 (33.3%)	16 (27.6%)
Tobacco history				
Never smoked	31 (23.1%)	15 (21.4%)	1 (16.7%)	15 (25.9%)
Ex-smokers	31 (23.1%)	12 (17.2%)	2 (33.3%)	17 (29.3%)
Current smokers	72 (53.8%)	43 (61.4%)	3 (50.0%)	26 (45.8%)

### Dysplasia in histopathological examinations

The histopathological diagnosis of dysplasia according to the socioeconomic characteristics is shown in Table 1. Of the 134 participants, 70 were histopathologically diagnosed with dysplasia, 58 were diagnosed with others, which were more likely to be benign, and 6 were diagnosed as oral cancer (squamous cell carcinoma). The mean age of participants diagnosed with oral cancer (62.45 ± 13.07 years) was higher than that of both those with dysplasia (58.71 ± 11.77 years) and others (53.34 ± 13.7 years). In addition, men were predominant in all three groups diagnosed with oral cancer, dysplasia, and others (83.3% vs. 81.4% vs. 94.8%). The proportion of patients with education status of senior high school or above was more in both the groups diagnosed with dysplasia and others (71.4%; 86.2%), but it was evenly distributed with those of lower education status in the oral cancer group. The proportion of participants living in the capital and out of it was evenly distributed among patients diagnosed with dysplasia and others; in the oral cancer group, the majority lived outside the capital. Regarding self-awareness, 90% of participants with dysplasia and 87.9% of those diagnosed as others were unaware of the lesion; but all oral cancer participants were aware of the lesion. For the para-habits, more participants diagnosed as others were never or ex-users for betel and tobacco (72.4% and 55.2%), and more participants diagnosed with dysplasia were current users of betel and tobacco (44.3% and 61.4%).

### Distribution of OPMDs and lesion sites

The results of COE are listed in Fig. 3. Among the 134 subjects, the predominant diagnosis was others, which was likely to be benign, and among all OPMDs, erythroleukoplakia was the most common (23/134, 17.2%), followed by thin homogeneous leukoplakia (21/134, 15.7%), and suspected oral cancer (10/134, 7.5%). Regarding the distribution of lesion sites, most of the lesions were located on the buccal mucosa (61/134, 45.5%), mandibular gingiva (16/134, 11.9%), and tongue (13/134, 9.7%).

### Association between severity of dysplasia and socioeconomic status

Results of the correlation between dysplasia and socioeconomic status are presented in Table 2. We noted a significant correlation between junior high school educational status and moderate/severe dysplasia (*P* < 0.001). Furthermore, lack of awareness was significantly associated with mild and moderate/severe dysplasia (*P* = 0.002). Regarding para-habits, current betel (*P* < 0.001) and tobacco use (*P* = 0.003) were strongly correlated with moderate/severe dysplasia.

**Table 2:** Association of demographic variables with the severity of OED

	Dysplasia		*P* value
	Mild	Moderate/severe	
	n (%)	n (%)	
	n = 49	n = 21	
Age (years)	58.61 ± 11.52	58.94 ± 12.62	0.915
Sex			0.406
Female	10 (20.4%)	3 (14.3%)	
Male	39 (79.6%)	18 (85.7%)	
Education			< 0.001
Junior high school	3 (6.1%)	17 (81%)	
Senior high school or above	46 (93.9%)	4 (19%)	
Residing			0.233
Capital	25 (51%)	8 (38.1%)	
Others	24 (49%)	13 (61.9%)	
Self-awareness			0.002
No	48 (98%)	15 (71.4%)	
Yes	1 (2%)	6 (28.6%)	
Betel history			< 0.001
Never or ex-used	36 (73.5%)	3 (14.3%)	
Current users	13 (26.5%)	18 (85.7%)	
Tobacco history			0.003
Never smoked	25 (51.0%)	2 (9.5%)	
Current smokers	24 (48.9%)	19 (90.5%)	

### ORs of the association between severity of dysplasia and socioeconomic status

Junior high school educational status had a high OR of 1, while senior high school or above educational status had an OR of 0.03 (95% CI 0.01–0.15; *P* < 0.001). The current betel users had a high OR of 6.57 (95% CI 1.17–37.0, *P* = 0.033) (Table 3).

**Table 3:** Odds ratio for demographic variables from TNOMSP relative to severity of OED

Characteristic	Mild dysplasia vs. moderate/severe dysplasia		
	Odds ratio	95% CI	*P* value
Education			
Junior high school	1	–	–
Senior high school or above	0.03	0.01–0.15	< 0.001
Betel history			
Never or ex-users	1	–	–
Current users	6.57	1.17–37.0	0.033

*Logistic regression with stepwise procedure

## Discussion

Several studies have demonstrated that socioeconomic status influences the development of OPMDs and OED, and the higher the socioeconomic status, the lower the risk of these conditions [13, 18–22]. A large population survey conducted in Taiwan showed a strong correlation between low education status and betel nut use habits, which may be explained by the labouring work in Taiwan with a cultural tradition of betel nut chewing. Moreover, the Taiwan study revealed that 25% of individuals with junior high school status were current betel chewers [23, 24]. This finding is similar to our result of overall dysplasia as we also showed that junior high school education status and current betel chewing were associated with a high risk of OED. Another study in Taiwan reported a 16.5% prevalence of OED among betel quid chewers [25]. Additionally, a hospital-based study revealed an OR of 8.5 (95% CI 4.4–16.2) for the development of oral malignancy among current betel quid chewers with low education status [11]. Moreover, another study in Taiwan revealed an OR of 1.27 (95% CI 0.93–1.75) for the development of OED among current betel chewers compared with non-chewers [26]. Furthermore, a case–control study reported an adjusted OR of 17.43 (95% CI 1.94–156.27) for the occurrence of leukoplakia due to betel nut chewing and smoking [12]. The above studies raised concerns about the increasing risk of malignant transformation in current betel chewers. Our present study also showed a high risk of developing moderate/severe dysplasia among those in the high-risk group (low educational status and current betel chewing).

Initiated in 1985, TNOMSP gradually scaled up to the national level and targeted the high-risk group [16]. High-risk individuals may be defined as tobacco users and betel chewers [27–29]. Approximately 90% of mortality resulting from oral malignancy in Southeast Asia occurs among individuals with para-habits, underscoring the need for efficient resource allocation to the high-risk group [30]. Moreover, oral cancer is one of the leading causes of death in adolescent males in Taiwan, with the overall 5-year survival rates for I–IV stages of oral cancer reducing from 70 to 10% [31]. Therefore, early prevention and correct diagnosis of OPMDs are needed to improve patient outcomes.

Most OPMDs are asymptomatic and rarely noticed by the patients, which is also evident in our present study as 85.1% of the participants were unaware of the lesion. The global prevalence of OPMDs is approximately 4.47% and is considered to be higher among Asian males [32]. Male predominance was also observed in our study. OPMDs mainly occur on the buccal mucosa, gingiva, tongue, and floor of the mouth [33–35]. Similar findings were reported in this study, with a majority of the lesions occurring on the buccal mucosa, followed by mandibular gingiva and tongue. Our study findings are also comparable to those of a recent large population-based study conducted in southern Taiwan [34]. Regarding the type of OPMDs in our study, erythroleukoplakia (17.2%) was the most common, followed by thin homogeneous leukoplakia (15.7%). When all types of leukoplakia, including non-homogeneous, thick, and thin homogenous leukoplakia, were included in the same group, the prevalence was 24.6%, and this finding is comparable to that of another large population study conducted in Taiwan [35].

Histopathological diagnosis is necessary for clinical grading of pathologic changes in OED and aids decision-making in managing lesions. According to the WHO guidelines, the standard grading system of OED is mild, moderate, and severe [36]. A recent meta-analysis evaluated the malignant transformation rate of mild vs. moderate/severe oral dysplasia. A total of 92 papers were selected and included in the final analysis; the mean follow-up ranged from 12 months to 20 years. Among them, 10 studies differentiated clearly between mild, moderate, and severe dysplasia. In summary, when comparing the risk of malignancy development with OED in moderate/severe dysplasia vs. mild OED, there was a greater risk for malignant transformation in moderate/severe dysplasia, with OR of 2.4 (99% Cl: 1.5–3.8). Further, mild dysplasia had an annual malignant transformation rate of 1.7%, while severe dysplasia was 3.57%, which were both statistically significant [37].

However, some have proposed a binary grading system and encouraged following pathological research [8, 38]. A recent smaller scale meta-analysis included 629 lesions from four different studies and yielded a six-time increased odds of malignant transformation in high-risk lesions over low-risk lesions OR of 6.14 (95% CI 1.18–15.3) [39]. In our present study, we categorised our cases into mild or moderate/severe dysplasia based on our current practices. Individuals with moderate/severe dysplasia undergo surgical excision of the lesions. The underlying reason for surgical excision is the necessity of further sampling, which can investigate the adjacent possible occult malignancy [1]. For mild dysplasia, a follow-up system is recommended, and the patient can undergo less invasive interventions, including cryotherapy, chemoprevention, and photodynamic therapy, which have shown promising results in preventing the malignant transformation of OED [40, 41]. Furthermore, OED has a significant malignant transformation rate. Although several studies we have mentioned suggested surgical excision for proper management of moderate/severe dysplasia, the long-term follow-up period should be less than every 6 months, repeated biopsy should be considered, and the absence of OED in clinical OPMDs diagnosis should not exclude the malignant potential [1, 8, 15, 37, 38]. Finally, even though some OPMDs with or without OED resolve spontaneously over time, the question remains whether the risk of transformation exists in OPMDs and OED after spontaneous resolution or surgical intervention, with or without para-habit cessation [42–44].

However, this study had several limitations. First, TNOMSP focuses on high-risk individuals aged > 20 years; thus, underage individuals with para-habits need further investigation. Second, the principle design of TNOMSP excludes the general population with OPMDs. Third, our study was conducted under a retrospective study setting and, our result was obtained from a limited diagnosis setting; a different judgement is hard to include in the study. Therefore, a large-scale investigation and comparison of individuals with para-habits and the general population should be considered with multiple specialties, and long term follow up with repeat biopsy may be consider in future research.

## Conclusions

In our study, we reported a high correlation between severe dysplasia and low education status, and current betel use. The socioeconomic status, poor self-awareness, and para-habits history of the patients with OED should be evaluated to identify high-risk individuals. This retrospective study lays the foundation for further investigation of the socioeconomic status and para-habits associated with OED in different regions across Taiwan with a larger population, as well as the evaluation of the efficacy of TNOMSP.

## Data Availability

The datasets used and/or analysed during the current study are available from the corresponding author on reasonable request.

## Abbreviations

CI: Confidence interval
NDMC: National Defense Medical Center
OED: Oral epithelial dysplasia
OMPD: Oral potentially malignant disorder
OR: Odds ratio
TNOMSP: Taiwanese Nationwide Oral Mucosal Screening Program
TSGH: Tri-Service General Hospital
